# The ethics weathervane

**DOI:** 10.1186/s12910-015-0054-4

**Published:** 2015-09-04

**Authors:** Bartha Maria Knoppers, Ruth Chadwick

**Affiliations:** Centre of Genomics and Policy, McGill University, 740 Ave Dr. Penfield Suite 5200, Montreal, QC H3A 0G1 Canada; Centre for Social Ethics and Policy, Manchester Law School, Williamson Building, Oxford Road, Manchester, M13 9PL UK

## Abstract

**Background:**

Global collaboration in genomic research is increasingly both a scientific reality and an ethical imperative. This past decade has witnessed the emergence of six new, interconnected areas of ethical consensus and emphasis for policy in genomics: governance, security, empowerment, transparency, the right not to know, and globalization.

**Discussion:**

The globalization of genomic research warrants an approach to governance policies grounded in human rights.

**Summary:**

A human rights approach activates the ethical principles underpinning genomic research. It lends force to the right of all citizens to benefit from scientific progress, and to the right of all scientists to be recognized for their contributions.

## Background

In 1994, the authors of this text exposed what they analyzed to be the ethical and legal norms underlying the Human Genome project from December 1989 to July 1994 [1]. We found several areas of international consensus centered on autonomy; privacy; justice; equity; and, equality, which out of respect for human dignity “could serve to harmonize eventual national regulation” ^pg. 2035^. We considered it urgent to have some codification in an international instrument and hoped that UNESCO would lead in this direction. In 1997, UNESCO adopted its *Universal Declaration on the Human Genome and Human Rights* [2].

We revisited these principles two years after the publication of the sequence map of the human genome in 2005. While still prominent, the principles were complemented by more social and communal concerns and trends in ethics that we identified as: reciprocity; mutuality; solidarity; citizenry; and, universality [3]. We concluded that “ethics does not consist of a static set of theories or principles that can be unproblematically ‘applied’ to new situations” ^pg.77^, and that “[t]here might not, and cannot, be universal norms in bioethics, as emerging ethical norms are as ‘epigenetic’ as the science they circumscribe” ^pg.78^.

A decade later, we again attempt to discern movement in international ethical norms. We posit that another six areas of complementary ethical consensus and emphasis have either emerged or moved to centre stage. They are: governance; security; empowerment; transparency; the right not to know; and, globalization. Two points need to be emphasized. First, these areas are not independent but rather intertwined. Empowerment and transparency, for example, are clearly connected. Second, the areas of ethical interest here are not specific to genomics only. Issues of security, most obviously, are at stake in a number of different contexts, and in some cases it is helpful to have regard for those contexts to further illuminate what is happening in genome-ethics. These developments inspired the adoption of a human rights approach manifest in the *Framework for Responsible Sharing of Genomic and Health-Related Data* (*Framework*) [4] of the Global Alliance for Genomics & Health (GA4GH) [5]. It is important to note that the *Framework* adopted such an approach due to the more universal and actionable character of human rights. Calling on article 27 of the *Universal Declaration of Human Rights* [6] and article 15 of the binding *International Covenant on Economic, Social and Cultural Rights* [7], the latter signed and ratified by 164 countries, the *Framework* envisages responsible data sharing founded on the right of all citizens to benefit from science, and the right to be recognized for one’s intellectual contribution.

What does a global momentum centered on activating these human rights reveal about the evolving international ethics landscape? The following sections describe how six areas of consensus are informing policies of genomic governance in this regard.

## Discussion

### Governance

Gottweis et al. defined governance as “[a] multifaceted compound situation of institutions, systems, structures, processes, procedures, practices, relationships and leadership behaviour in the exercise of social, political, economic, and managerial/administrative authority in the running of public or private affairs” [8]. Governance can further include professional guidelines in addition to procedures and practices. It has, to some degree, become an umbrella term for different ways of ensuring that the interests of affected parties are appropriately safeguarded.

The emergence of biobanks attests to the impracticalities of obtaining individual consent for every future use of stored data and samples. Nowhere is the gradual shift in emphasis from individual consent to governance better illustrated than in the adoption by the United States National Institutes of Health (NIH) of its Genomic Data Sharing (GDS) Policy on August 27th, 2014 [9]. The GDS Policy expects researchers to: “seek consent from participants for future research uses and the broadest possible sharing”; deposit such data in NIH designated repositories; and use Trusted Partners via contractual mechanisms for storage or for analytical tools. The NIH provides elaborate guidance as well as the creation of DACs (Data Access Committees) with a notification system for any alleged misuse or mismanagement. Moreover, “secondary users in violation of the Policy or the Data Use Certification may face enforcement actions”. De-identified data for the NIH will now be considered controlled data, and users must be approved by a DAC. Controlled access illustrates an approach to governance already tried and tested in international consortia such as the International Cancer Genome Consortium (ICGC) [10]. Data in open repositories such as the 1000 Genomes Project continue to be publicly available, however these are not individual level data [11]. Under the GDS, appropriate governance with oversight lines of accountability and sanctions counterbalance the trust of participants who provide broad consent for future unspecified research uses of their data and samples.

### Security

While privacy remains a primary concern for the use of genetic or genomic data—especially now that re-identifiability of previously anonymized participants cannot be ruled out [12, 13] with the arrival of Big Data—the locus of privacy protection is shifting to security. In essence, security is the state of being free from danger or threat and is a concept that applies in a number of fields. Food security relates to the availability of a sustainable food supply; airport security deals with threats to safety among travelers and personnel. The central issue in the context of data security is preventing unauthorized access, which includes but goes beyond individual privacy concerns.

The challenges arising from technological change have led some to portray the situation we currently face as a trade-off between privacy and security, but the situation is more complicated than that. There is a public (and not only an individual or group) interest in privacy as well as security, and security itself may be necessary to safeguard this public interest.

The security issues pertaining to personal data present some of the most complex challenges of the current decade. Concerns over the security of personal data, particularly in Europe following the National Security Agency scandal, and together with ever- increasing cloud computing for large datasets, make data security a priority [14]. The travails and tribulations of the proposed European Data Protection Regulation [15] attest to the concerns of policymakers and the public about unauthorized access to, and use of sensitive data. The proposed stringent protection of sensitive data has led to much discussion in Europe over the need to create exemptions for research using health data (including genetic data) [16]. Safe havens for data deposit and use have been proposed as part of security with the following common attributes: data maintenance and release must be socially acceptable and appropriate, data must be veritable, and data must be safe and secure [17].

### Empowerment

In 2005, we described the growing trend to incorporate public consultation, engagement or involvement in research under the rubric of “citizenry”. While a ubiquitous phenomenon, engagement is not necessarily a panacea. In light of noticeable moves toward evaluating engagement activities, what difference do they actually make to policy?

Today, empowerment of individuals goes beyond institutionalizing strategies for including the public. The mechanisms of public engagement have undergone a series of transformations. The move towards upstream engagement promised the introduction of lay expertise into issues of scientific governance and research agenda-setting. This move was a reaction against the drawbacks of programmes directed at improving public understanding of science, which have been described as a “false dawn” [18] where sponsor interests dominate dialogues. Watermeyer introduces the idea of the ‘polylogue’ in place of ‘dialogue’, which is facilitated by the use of online activities in public engagement [19]. The polylogue differs from the dialogue in that it not only multiplies the number of conversationalists, but also the number of conversations occurring and regenerating at any one time. Thus Web 2.0 may appear to offer a deeper sense of the democratization of science [20]. Another advantage of the polylogue is that it “follows more faithfully the pursuit of the scientist in asking new questions, rather than the policy maker in demanding new solutions” [18]. It also has associated risks, however. An over-abundance of subjective points of view create radical uncertainty, and introduces uninvited conversations into public policy debates. Wikipedia has demonstrated the added problem of crowd sourcing, and its production of (questionable) knowledge claims.

Another dimension of citizenry is that patients network to make research endeavors their own. Organizations in the U.S. such as Genetic Alliance [21], PatientsLikeMe [22] and the Patient-Centered Outcomes Research Institute [23] serve as examples. Another example is the Personal Genome Project [24], where individuals can put their whole genome on the web. The willingness to publicly share personal (phenotype; socio-demographic etc.) and genetic health data on the web, or to be contacted by researchers for possible inclusion in trials typifies this movement. Patient groups are concerned with a more efficient translation of research into the clinic—an expanding area of research in its own right. What are the challenges, barriers and bottlenecks that impede the process of translation? These include ethical challenges in addition to ensuring adequate resources and preparedness of personnel.

### Transparency

Transparency refers to the ways in which activities are carried out openly. Patient information is accessible not only to interested parties, but also to the public at large. Based on recent figures from the Transparency Initiative first launched by the European Medicines Agency, a proactive clinical trial data sharing policy was proposed [25]. Furthermore, the work of the All Trials petition [26] and GlaxoSmithKline [27] led to a portal for anonymized data release for clinical trials, and are both indicative of greater access to industry data. Today, such available data includes completed clinical trials whether published or not and whether successful or not. The data are, however, limited to the scope/disease of the original consent even when anonymized. As of September 2014, 10 companies are listed as users of the system. Of the 119 research proposals submitted to the website between May 7th, 2013 and March 31st, 2015, 83 were approved or approved with conditions [28].

Research proposals are checked to make sure the information is complete and that they meet the informed consent requirements of this initiative and of the sponsor. They are then sent to the Independent Review Panel. Panel members receive a brief research proposal including analysis and publication plans once it has been processed for scientific credibility. The Panel accepts or rejects proposals based on scientific merit, researcher qualifications and management of conflicts of interest. In early 2015, the Institute of Medicine (IOM) lent its support to data sharing in clinical trials [29]. The IOM called for a multi-stakeholder effort to foster a culture of responsible data sharing, with a focus on improved infrastructure, technology, operational management, and funding.

### Right not to know

Access to information is not always empowering. While the argument for a ‘right not to know’ is not in itself new, it is being deployed in novel ways. There has been a tsunami of data (still mostly indecipherable) with the arrival of next generation sequencing technologies (NGS). Due to the very nature of NGS, the data revealed are often beyond the research objectives. Secondary, rather than incidental findings may be a better term for such data as a result [30, 31]. Indeed, we posit that in the near future more emphasis may well be placed on asking individuals what they do not wish to know when they consent to research or diagnoses that utilize NGS.

There continues to be discussion about how a right not to know should be conceptualized. Graeme Laurie in the second edition of *The Right to Know and the Right not to Know,* argues that while autonomy underpins the right to know, the interests at stake in the right not to know are best construed as privacy interests, except where an individual has expressed a prior wish not to know [32]. The right not to know finds its ‘genetic’ origins in the Human Genome project, and is enshrined in two international documents adopted in 1997 by UNESCO [2] and the Council of Europe [33]. The right not to know has only recently appeared in two U.S. policies that recognize its legitimacy in genetic disclosure [30]. Although both documents contoured the right not to know, there are particular contexts where this right requires immediate further delineation, namely in pediatrics. The international pediatric platform of the Public Population Project in Genomics, for example, recommends that parents not be allowed to exercise their right not to know when the findings reveal a clinically significant condition that is treatable or preventable during childhood [34].

### Globalization

In this article we are concerned not so much with globalization as an economic and political phenomenon, but with the global dimensions of ethics. When we refer to the ways in which ethical issues are global we mean one of two things: either that issues have a global reach (for example foreign aid, where actions taken in one part of the globe impact another) or that they are global in themselves (such as climate change or pandemics). In the past 10 years, the global dimension of issues in genomics has become more important. Speaking of the human genome as the common heritage of humanity, even in a symbolic sense, suggests genomics is global at the level of the genome. Specific issues in the first sense are now more central, namely the transfer of samples and data across borders. Data sharing between different biobanks is necessary in order to achieve sufficient statistical power to underpin significant results [35]. This development clearly challenges the principles discussed above, notably privacy and security but also accentuates the procedural inefficiencies of research ethics review. If research is increasingly global, a system for mutual recognition of substantial equivalency of ethics review and of oversight in different jurisdictions is key for collaborative science generally, but genomics and multi-centred clinical trials specifically [36]. Concerns about whether samples can be appropriately protected in different jurisdictions raises questions about the possibility of ethics review harmonization. In other words, is there enough commonality as to the governance mechanisms needed for review? The GA4GH is moving towards meeting this challenge.

## Summary

We posit there is a movement towards emphasizing governance, security, empowerment, transparency, the right not to know and globalization in the contemporary ethics of genome medicine. Recently, this phenomenon of “ethical-globalization” motivated a more politically actionable human rights approach to the above areas as demonstrated in the data sharing *Framework* of the GA4GH and others. International interoperability and harmonization promote and facilitate the principles we describe, but there is no doubt that more profound structural changes are required to buttress and realize these principles. Clear systems of governance, public trust in data security, personal empowerment and the responsibility it brings re “knowing” (or not) as well as transparency of research outcomes are to be welcomed. The human rights of all citizens to benefit from scientific advances, and of scientists to be recognized for their contributions will only become truly actionable if they underpin the very raison d’être of responsible science.

## Abbreviations

UNESCO: United Nations Educational, Scientific and Cultural Organization
GA4GH: Global Alliance for Genomics & Health
NIH: National Institutes of Health
GDS: Genomic Data Sharing
DAC: Data Access Committees
ICGC: International Cancer Genome Consortium
IOM: Institute of Medicine
NGS: Next generation sequencing technologies
